# Paradox of Modern Pregnancy: A Phenomenological Study of Women's Lived Experiences from Assisted Pregnancy

**DOI:** 10.1155/2015/543210

**Published:** 2015-05-03

**Authors:** Fahimeh Ranjbar, Mohammad-Mehdi Akhondi, Leili Borimnejad, Saeed-Reza Ghaffari, Zahra Behboodi-Moghadam

**Affiliations:** ^1^School of Nursing & Midwifery, Tehran University of Medical Sciences, Tehran 1419733171, Iran; ^2^Reproductive Biotechnology Research Center, Avicenna Research Institute, ACECR, Tehran 196151177, Iran; ^3^School of Nursing and Midwifery, Iran University of Medical Sciences, Tehran 1996713883, Iran

## Abstract

The purpose of our study was describing the meaning of pregnancy through Assisted Reproductive Technologies (ARTs). A qualitative design with hermeneutic phenomenology approach was selected to carry out the research. Semistructured in-depth interviews were conducted with 12 women who experienced assisted pregnancy. Three themes emerged from women's experience including finding peace in life, paradoxical feelings, and struggling to realize a dream. We concluded that pregnancy is the beginning of a new and hard struggle for women with fertility problems. The findings of our study resulted in helpful implications for the health care professionals managing assisted pregnancies.

## 1. Introduction

Infertility treatment has quite many physiological and psychological effects on people and getting pregnant does not necessarily mean an end to these problems [1]. Treatment via Assisted Reproductive Technologies (ARTs) is quite an emotionally and physically difficult process [2]. The specific stressors associated with this treatment are beyond the positive pregnancy test [3]. Couples who become pregnant after ARTs cannot easily overcome the negative feelings related to infertility and are different in a number of personality dimensions and affective reactions toward pregnant women having become pregnant naturally [4, 5]. In pregnancies resulting from ARTs, women sometimes experience new stresses, uncertainty or fear about pregnancy [6]. Signs of increased anxiety are found more in mothers and spouses who have experienced high infertility stress. Those couples who become pregnant through ARTs are more anxious in the early stages of pregnancy than those who conceive naturally. These couples are more fretful about pregnancy loss [7]. With each failure and even success a feeling of not becoming a mother will bother them again [2]. Although these techniques are known as medical technology, sociocultural norms are considered a very important factor in its acceptance. Sociocultural norms may act as a barrier or even cause later problems [8]. Understanding the context and complexity of such pregnancy is so important for the health care professionals working with these women [9]. As more families are seeking pregnancy using assisted reproductive techniques, more research is needed to explore the concept of assisted pregnancy [10]. ARTs have become increasingly popular in Iran because of high prevalence of infertility. More than 20% of Iranian couples experience infertility during their reproductive life. Currently, more than 75 clinics provide ARTs in all types [8, 11, 12]. Despite the fact that a considerable body of literature has focused on infertility in qualitative researches in Iran [13–17], less is known about the sociocultural problems and the need of Iranian women who have experienced assisted pregnancy. The purpose of our study was to explore how women make sense of assisted pregnancy in Iranian culture and context.

## 2. Method and Material

Qualitative research has been deemed as a valuable tool for collecting and analyzing data in complex health and social issues [18]. Hermeneutic phenomenological method as described by van Manen was employed for our study. This approach is the combination of descriptive and interpretive phenomenology [19].

### 2.1. Participants

The participants were Iranian infertile women who experienced pregnancy through ARTs. Using purposive sampling, 12 married women were recruited in the study. The inclusion criteria were the first and single pregnancy via ARTs, history of primary and female-related infertility, and the ability to speak Persian fluently. Exclusion criteria were history of miscarriage, assisted pregnancy through donated gametes, severe complication during pregnancy, and fetal abnormalities. Participants with female etiology of infertility including polycystic ovarian syndrome, blocked tubes, fibroids, decreased ovarian reserve, and immunological factors participated in our study.

### 2.2. Data Collection

The study was conducted in Avicenna Infertility Clinic in Tehran, Iran. The setting was a semipublic and nonacademic referral center affiliated to the Academic Center for Education, Culture and Research (ACECR). The first inclusion criterion was based on the patients' medical records. The convenient time for the interview was suggested by participants. All interviews were conducted by the first author in a private room in the clinic.

Most of the participants were from cities other than Tehran and all interviews were conducted in the infertility center. Data redundancy was recognized after interview with 10 participants, but two other participants were interviewed for assurance. Finally, 12 women were recruited in the study.

Semistructured interviews were performed in 17 sessions and during a six-month process from August 2013 to February 2014. Data were collected by in-depth interviews with approximate duration of 30 to 60 minutes. Participants were asked to talk about their experience with assisted pregnancy after infertility. Interviews began with questions about their initial feelings and perception toward pregnancy. The interviewer summarized participants' speaking at the end of sessions for data confirmation. Participants were encouraged to provide new information. Participants were free to ask questions or leave the rest of interview at any time. We wrote field notes after each interview and all interviews were recorded and transcribed verbatim immediately and then converted to Rich Text Format for MAXQDA 10 software (VERBI GmbH, Marburg, Germany) to facilitate data management.

### 2.3. Data Analysis

Data were analyzed using van Manen interpretative phenomenological strategies. According to van Manen, the selective and detailed or line-by-line approach was used to isolate thematic statements. van Manen has suggested the following 6 inseparable steps for researchers which are used in our study:

1. Focusing on the phenomenon that deeply interests us and makes our minds engaged: in this regard, the researcher's mind was constantly engaged with this question: what is the meaning of assisted pregnancy after infertility?
2. Exploring the phenomenon as something alive rather than what we conceptualize: to make the researcher interact with the main experience, women who actually had experience with this phenomenon were invited to our research.
3. Reflecting on the themes that reveal inherent characteristics of the phenomenon: researchers must constantly ask themselves what the nature of experience with assisted pregnancy after infertility is. In answering this question, inherent themes will be understood.
4. Describing the phenomenon with the art of writing and rewriting: phenomenological analysis is primarily an exercise in writing and thereby the researchers can achieve the meaning of experience through the practice. The researcher should try to reflect experience in such a way that the reader feels that he/she has experienced the phenomenon under study and is able to have the same result about its meaning.
5. Establishing and maintaining a conscious connection with the phenomenon: the researcher should be preoccupied with the lived experience of pregnant women via ARTs. Creating a strong connection between the text and the phenomenon and using rich and in-depth descriptions of the findings reduce the likelihood of deviation from the main path.
6. Balancing the research context by considering the parts and the whole: in the last step, both the whole and contextual data are considered and the relationship of each part in the formation of phenomenon is examined [20].

### 2.4. Trustworthiness

Lincoln and Guba explained that credibility, confirmability, dependability, and transferability ensure the rigor in qualitative research. In order to achieve credibility in our research, maximum variation sampling, immersion and long engagement of researcher in the field, persistent observation, data triangulation, peer-checking, and member-checking were done. Experience of the authors in the field of infertility, ARTs, prenatal care, and qualitative research methods enhanced the confirmability. The data analysis process has been approved by all members of the team. All interviews were recorded and transcribed immediately by the first author to ensure dependability. The first author wrote her preunderstanding regarding pregnancy through ARTs prior to study and made efforts to bracket them in data analysis process. So as to meet transferability, the context of information collection is fully described.

### 2.5. Ethical Consideration

Ethics Committee of the Tehran University of Medical Sciences and the Avicenna Research Institute approved the research proposal. Participants were assured about confidentiality of their responses. The purpose of the study was explained verbally and written consent was obtained from all participants. The participants were ensured that audio files and their transcriptions were stored anonymously and separated from consent forms.

## 3. Results

The mean age of participants was 29.2 years at the time of interview. Only two participants were employees. The treatment method resulting in pregnancy was ICSI for all participants. Women's characteristics are summarized in Table 1.

### 3.1. Emergent Themes

After data analysis using MAXQDA software and reviewing over 700 meaning units, three themes were generated: finding peace in life, paradoxical feelings, and struggling to realize a dream (Table 2).

### 3.2. Finding Peace in Life

Women became relieved by this new experience. They ultimately got pregnant after struggling with infertility and their stress level declined. The fear that they would never become pregnant anymore was alleviated. Subthemes within this theme were feeling of becoming a mother, security and power, self-confidence, and finding meaning in life.

Participants showed more affection to the fetus. They described the feeling of becoming a mother as a new and the best feeling in the world. Some women had the affection even before embryo transfer and had a good feeling after seeing the embryos on the screen. Women had a sense of responsibility toward their fetus especially after the first trimester of pregnancy. They talked, read, and sang to their baby and were very excited when their babies kicked. One of the participants described the sense of being a mother as below: “Something is growing in your body; you are the person giving life, sense and peace to that. It is just a flower to which you are giving water and light. You give everything to that and if you take care and give it everything, you will have a very beautiful, healthy, and fragrant flower.”

Sense of power and security was the greatest achievement of pregnancy for these women. This sense was connected with satisfying the husband, improving marital satisfaction, and keeping marriage alive. They did not want infertility to ruin their marriage and they frequently burst into tears when describing the strong feelings of their spouse about children. Some narratives, such as “My husband was jealous of others' babies… I thought that his attention toward children would become more and more… He played with babies and kissed them… He did not say anything to me about infertility, but I knew that he loves children a lot,” were common in all the interviews. One of the participants declared her happiness because of satisfying her husband: “My husband was so upset that we would never have a child, and I was sad because of him (bursting into tears). I am glad he achieved his wish.” Another participant said: “My husband is more motivated to live with me and my new baby now. I was sure that he was not happy before and maybe he thought we would be separated finally.” Marital satisfaction had also increased with the change in husband's behavior and his support during pregnancy. They said that their relation would become better and pregnancy or the presence of a child can bring them closer at times. One participant stated the following: “From the time I got pregnant it seems that there is a sense of happiness in him. He takes care of me more now. He asks me whether the baby moves in my belly. How do you feel now? Does anything bother you?”

The extra attention that the society draws to pregnant women results in a feeling of self-confidence and self-worth. They got rid of the stigma of infertility and nothing was missed in their life anymore. One participant declared her self-confidence with these sentences: “I feel more confident in my life now; it was a nice experience for me. While I was walking in the streets I thought all the people were looking at me.” One woman stated how pregnancy was a means of escaping from infertility stigma: “In every party or religious ceremony people came to me and said we bring this for you because of your pregnancy. The feeling and opinion of people toward me has changed now.” Another informant said: “Maybe it is not the case, but before pregnancy I thought something has been missed in my life.” One woman mentioned: “I think everything is peaceful now. I have the authority to make decisions and to have the last say. All the attentions are on my child and me.”

Women remarked finding meaning in life. They found hope in life and it was time to say goodbye to isolation, loneliness, and monotonous life. For example, one of the women discussed: “We are more hopeful. We look at life in another way. Our life became really monotonous. Now it is more exciting.”

### 3.3. Paradoxical Feelings

Participants have experienced mood swings and irritability during pregnancy. The joy, fear, hope, and uncertainty are some paradoxical feelings. Women were excited and scared after the shock and disbelief. One woman described the paradox of joy and fear as follows: “They said ok madam you are pregnant. At that time I had a feeling that I could neither cry nor laugh. I did not know what to do. You know? I was a little bit surprised…It was positive…I was so happy because of the positive pregnancy test, but suddenly I said to myself what if I have an ectopic pregnancy.” Another woman stated the paradoxical feeling as follows: “It is obvious that it is a stressful situation. It is really difficult. Will this pregnancy last? I am really worried whether something happens until the day of childbirth… There are a lot of beautiful fantasies like embracing my child and of course there are some stresses.”

Despite the fear and joy, the women's stories revealed that they also experienced hope and uncertainty. There was a great deal of uncertainty, especially about losing the pregnancy. They seemed to be caught between fertile and infertile worlds. Some issues, such as hCG level and its titration, ectopic pregnancy, hearing the baby's heartbeat, miscarriage, birth defects, feeling the baby's kicks, and premature labor, were almost paralyzed at times. One woman stated: “The paradox is that sometimes you say to yourself ok, I have passed all the steps, I did what the doctors recommended and there is no need to be upset and worried, but you think to yourself that the other woman also did all these steps and it was her sixth time. Will this pregnancy last? What if I also become like that woman.” Sometimes women were not convinced that they were pregnant even after a positive pregnancy test. They described it was so difficult to believe a positive pregnancy test after experiencing infertility. For instance, one of the participants said: “I did not believe that. I thought to myself if it is possible for me to have an embryo. I said they have made a mistake and I am not pregnant or I said the embryo does not have heartbeat.” They had doubts about being pregnant and they did not speak with certainty about their pregnancy. Many times the fear of miscarriage changed their decision to reveal pregnancy to others. One participant said: “I have some friends and during these 2 or 3 weeks they came to our house, but I did not say anything to them since I was not sure about my pregnancy. I intended to wait at least for two months…Nothing is clear right now. The doctor could not see any special thing via ultrasound.” Women were concerned about miscarriage and some of them reported that they had checked for bleeding or spotting every time they went to the bathroom. For example, one participant said: “The first three months passed with difficulty and since we expected some bad experiences, we got worried soon. I always checked my underwear in bathroom to see if there is any problem. Two weeks ago it seemed that I had some brownish spots. It was the time when I just sat and started crying loudly.”

Sometimes uncertainty did not allow some women to start to feel attachment to their fetus. The majority of women confessed that they had postponed shopping for baby clothes and supplies until the third trimester. One participant remarked: “Now I am in doubt whether I should bond with her. What if something happens to that or a problem occurs? I am a little bit stressed out.” Some found it hard to believe that they might actually end up with hugging a baby. One woman said: “I cannot bond with my unborn child, I am afraid I could not carry it into the third trimester. They had to bear the brunt of the impact.” Contrary to the sex of the fetus, the well-being of the baby is a usual concern and priority. At times, women were eased only by an ultrasound showing a healthy baby. One participant said: “I repeat this sentence to myself what if the doctor says there is a problem. It was killing. Until the day of ultrasound, my heart will stop beating since I am really stressed out.”

### 3.4. Struggle to Realize a Dream

The lived experience of the women who became pregnant through ARTs is a process of struggling to realize a dream which includes going through all difficulties, change in life style, and spirituality. The participants started a deliberate attempt to become a mother. They endured all the challenges of ARTs including the multiple, long, and difficult cycles of treatment, its high costs, pain and discomfort, and finally the worst kind of waiting to fulfill their dream. They had to go through the difficult physical, emotional, and financial treatment and did not give up their dream until they put it into reality. They did not complain about the loss of freedom and loss of attractiveness during pregnancy or common physical changes and discomforts in early pregnancy. For example, one participant said: “My situation was very difficult because I was struggling to achieve something; I had to start receiving the everyday shots. It was really difficult at first, but it is pretty ok now. Pain had no meaning to me because of baby.”

Financial stress related to incomplete health insurance coverage in Iran was the other concern. Sometimes finding the drugs was difficult for women struggling with fertility problems. One participant remarked: “I do not like being a burden on other people. In the time of infertility sometimes the expenses became so high that we did not afford to pay for them. My husband borrowed some money from someone and when I did not become pregnant, it was a burden on my husband's shoulder.”

One woman remarked that despite her discontent, she has accepted help from her mother-in-law: “My husband is in another city for work and it is about six months that I am living in my mother-in-law's house. They are really kind to me, but it is difficult for me. This is not their responsibility to take care of me. Sometimes we have to bear something not for the sake of ourselves but for the sake of our children.”

Announcing the pregnancy to others was even a more complex problem. Fear of miscarriage was the most common reason for not sharing pregnancy news early. They were also worried about the negative community reactions to the test tube babies. Sometimes women managed not to reveal the assisted pregnancy. In case of disclosing assisted pregnancy, women were stigmatized by their in-laws, relatives, and friends. In case of prolonged infertility, pregnancy has been repeatedly denied by others in early stages and people asked about the origins of the child or sometimes thought that these women used the donated gametes. One woman said: “in the area that we live, people think that in assisted pregnancy the embryo belongs to someone else. I said to them that I got pregnant naturally, but they thought that I had implanted another one's embryo.”

Women experienced a wide variety of adjusting behaviors such as decline in physical activity, sexual intercourse, change in diet, and avoiding high-risk behavior. Now all have gone aside, and the focus is just on the baby. One participant described her adjustment to pregnancy after infertility as follows: “Before that we traveled a lot, but during the past 6 or 7 months we have not travelled at all. I myself stayed at home most of the time and so did my husband. We had to put aside many things that we did before in order to have a child.” Another woman mentioned: “I had a headache for about 3 or 4 days, but I did not take any kinds of tablets due to the fear of its side effects on baby. When I made a phone call to my doctor, she told me to take a pill but I did not.”

The results illustrated that thanks giving and trust in God, reading holy Quran, and praying for their fetus and other infertile patients are the most cited sources of spiritual experiences. All the participants were Muslim. One participant declared the trust in God as follows: “When I became desperate, there was a feeling that told me to move on and not to be frustrated. There is always the one who can help you from the above. I was hopeful. The day that I went for the transfer I said oh God I just rely on you, do not send me back empty-handed from here.” Another woman remarked the sense of her inability to keep the fetus in this way: “Sometimes I feel that I can do nothing more myself. I read holy Quran and say prayers. I rely on the strength from God. When you see you cannot do anything more for yourself, you start asking help from God.” Some participants encouraged their friends with fertility problems to pursue the treatment process. Participants felt uncomfortable talking about their pregnancies with infertile friends who are still undergoing treatments. Sometimes participants were upset because of the treatment failure in their friends and also the financial problem that does not permit them to repeat ARTs cycles. One participant mentioned: “Many times I thought about those people who have the same problem as mine and cannot afford the expenses and I told myself what if all of them could come here.” Another woman said: “One of my friends did not get pregnant. I was really sad about her. Why should I get pregnant, but she does not. Her husband had told her that this is the last time. I spoke with her a lot, but she said she would not pursue.”

## 4. Discussion

Our study discussed how Iranian pregnant women undergoing ARTs make sense of their conditions. The meaning of assisted pregnancy manifested itself as finding partial peace in life. The findings of our study support previous research on women's lived experiences by assisted pregnancy. Pregnancy had given women a sense of shock and surprise consistent with what was found in the study of Toscano and Montgomery [3]. Also, Redshaw et al. noticed the sense of being complete among women who successfully became pregnant as one of the findings in their study [21]. In the present study, women were very relieved and pregnancy had given women a sense of peace especially because of the power and security in marriage. Most of the women had the risk of this treatment as the unequally shared burden against security in marriage. In patriarchal societies like Iran, women have been identified with their role as mothers, and sometimes they are coerced to be mothers [22]. Children are actually seen as the link between women and their husbands in Iran, and infertility threats marriage [23]. Some participants in the present study said that they had a friendly relationship with their husband before, but they were concerned that infertility may become a problem over time and they may lose their spouses in the future. They wanted to achieve a stable home and family. Consistent with the present study, Hasanpoor-Azghdy et al. found out that pregnancy can stabilize the women status in the family and community [14]. Some participants reported that pregnancy is an end to coldness in marital relationships and the end of the pressures from their relatives and in-laws. Women referred also to the more supportive role of husbands during pregnancy that caused strength and feelings of security in them. Such support was less during infertility treatment. These findings are similar to other studies in Iran and Taiwan [1, 23, 24].

Results showed that women experienced an increase in self-confidence. Although some studies reported similar self-confidence in assisted pregnancies in comparison with the control group, these studies also considered the increased confidence as pregnancy progresses [7, 25, 26]. Lower level of self-esteem and the feeling of personal deficiency have been reported in infertile women [3, 15]. In the present study, this feeling of lack disappeared in pregnant women.

The emotional pain women experienced when going through assisted pregnancy was hard. Most of the participants in the present study did not inform others of their pregnancy through ARTs. Majority of concerns related to pregnancy announcement were due to social stigma. Women with short infertility period were more successful in hiding assisted pregnancy and tried to keep their secret from others especially their in-laws. As discussed before, the most important duty for an Iranian woman is becoming a mother and infertility or assisted pregnancy can stigmatize her status in the community [23]. In the present study, the main reason for disclosing pregnancy through ARTs was to receive support during pregnancy. Providing counseling with regard to disclosure issues has been emphasized in pregnancy after infertility [5]. As women do not like to share their problem with relatives or people who are close to them, their privacy and confidentiality should be respected more in the prenatal visits. Providing some practical coping tips may be helpful in case of stigma.

The woman's emotions were often unstable. This is also in accordance with other studies stating the experience of joy, fear, uncertainty, ambiguity, and confusion during assisted pregnancies [1, 3, 24]. Uncertainty in bonding to the fetus was also visible in the present study. Mothers tried not to have strong emotional attachment with baby especially in early stage of pregnancy because of the fear of losing the baby. Uncertainty can remain until a healthy baby is delivered. Difficulty in bonding to the fetus has been documented before [27], but there is a controversy and some researchers believe that the attachment to the fetus is similar to other pregnancies [7]. Emotional support of couples who conceive through ARTs has been emphasized because of postnatal mood disturbance and difficulties in early mothering [4, 5, 9, 28, 29]. Participants need support from health care providers in adjustment to pregnancy to reduce the stress. Anyhow, health care providers should reassure the clients in counseling meetings that the bond between mother and fetus sometimes does not happen instantly after the positive pregnancy test and it may be extended until the second or third trimester.

Pregnancy is the beginning of a new and hard struggle for women with fertility problems. Women in the present study have to deal with the ARTs challenges to fulfill the dream of becoming a mother. Although some of the participants described their pregnancy as abnormal, they had no intention of adopting a child and wanted to have their own pregnancy experience. Similarly, in a study conducted in India, women tried to overcome the hardships and challenges despite the high physical, emotional, and financial difficulties and the low success rate of the treatment process. These women never thought about stopping their treatment also [30].

Women had to go through the change in their life style and spirituality which had been also reported in similar studies [1, 24]. Regardless of the fact that women's daily life had been strongly affected by pregnancy, they were not complaining about change in life style, resting at home, isolation during pregnancy, or elimination of all their freedom. These results were inconsistent with findings of other studies in which women were inconvenient about physical changes [1, 24]. Participants noted that spirituality helped them struggle with their challenges. Many of them were willing to help those having similar problems. Some studies have also looked at the role of spirituality as a protective factor during pregnancy [31]. As a result, we can support the client's spirituality to enhance the patient care.

## 5. Conclusion

The findings of the present study lead to a preliminary understanding of Iranian women's experiences from pregnancy through ARTs. This concept may be of much value to health care providers supporting women during an assisted pregnancy. Pregnancy is one of the hardest parts of realizing the dream of having a child for former infertile women. On the one hand, pregnant women get relieved and find more power and security in marriage. Nevertheless, women's emotions were often unstable and pregnant women with history of assisted reproductive techniques did not have a more positive experience. Pregnancy as a shocking surprise, a feeling of great relief, uncertainty about the outcome of the fetus, difficulty in pregnancy announcement, change in life style, and turning to religious beliefs had been documented in similar studies, but senses of power and security, marital satisfaction, and IVF stigma are some of the new issues in the present study due to the sociocultural differences. It seems that empowering pregnant women, handling stress, noticing attachment to their babies, managing paradoxical feelings, respecting women's confidentiality, reinforcing the coping strategies in case of stigma, and providing spiritual care to mothers should be construed as extra care in assisted pregnancies. As pregnancy may be influenced by the infertility treatment, the special care needed in an assisted pregnancy should be never taken for granted by health care providers. Health care providers should be well aware of the unavoidable effects of infertility on the experience of pregnancy. Developing new midwife roles and continued research in different contexts would enable health workers to provide a good care in assisted pregnancies.

### 5.1. Limitations of the Study

The two main limitations of the qualitative research methods are related to the issues of generalizability and replicability of the study [32]. In this respect we followed the purposive sampling approach and we also provide information about the context in which we were collecting the data to address some of these concerns. Anyhow all participants were recruited from one infertility center and their experiences might differ from those who became pregnant in other centers. Sadly enough, fewer women followed their pregnancy care inside the infertility center. Therefore, accessing to all of the women who got pregnant in this clinic and organizing the interviews in the last months of pregnancy were not possible.

## Figures and Tables

**Table 1:** Demographic characteristics of subjects.

Participant	Age	Level of education	Gestational age (week)	Marriage duration	Infertility duration (year)	The cause of infertility	History of treatment failure (*N*)	Ethnicity
1	33	M.S.	4, 8	5	5	Hypothalamic amenorrhea	—	Kurd
2	24	B.S.	5	3	1	PCO	IO (1)	Fars
3	30	HS	4	3	1	DOR	Io (3)	Fars
4	28	HS	20, 29	13	10	Tubal factor	IUI (1)	Fars
5	36	HS	13, 19, 28	15	14	Tubal factor	Io (3)	Fars
6	28	HS	20	9	5	PCO	ICSI (1)	Fars
7	30	B.S.	20	14	11	PCO, tubal factor	ICSI (3)	Kurd
8	26	B.S. student	16, 20	6	5	Fibroma	IUI (5)	Fars
9	32	HS	9	13	12	Immunological	IUI (5) ICSI (1)	Turk
10	25	HS	4	7	6	Immunological	ICSI (5)	Turk
11	28	9th grade	12	12	11	Fibroma and adhesion in uterus	IUI (1) ICSI (1)	Lor
12	31	M.A. student	28	7	3.5	DOR	IUI (3)	Turk

HS: high school, B.S.: Bachelor of Science, M.A.: Master of Art, and M.S.: Master of Science.

IO: induction ovulation, IUI: intrauterine insemination, and ICSI: intracytoplasmic sperm injection.

**Table 2:** Themes arising from women's perceptions of assisted pregnancy.

Theme	Category	Subcategory
Finding peace in life	Power and security	Satisfying husband
		Marital satisfaction
		Keeping the marriage alive
	Finding self-confidence	Feeling lack of something
		Being the center of attention
		Getting rid of the stigma of infertility
	Finding meaning in life	Escape from isolation and loneliness
		Getting rid of monotonous life
		Finding hope
	Feeling of being a mother	Bonding to the fetus
		Sense of responsibility toward the fetus

Paradoxical feelings	Joy and fear	Surprise
		Joy and Fear
	Hope and uncertainty	Uncertainty of being pregnant
		Doubt about the baby's health and safety
		Uncertainty to continue pregnancy to the end of the third trimester
		Uncertainty in bonding with unborn baby

Struggle to realize a dream	Enduring all challenges	Difficult physical, emotional, and financial treatment
		Stigma
		Seeking help out of desperation
	Change in the life style	Low physical activity
		Adaptive behaviors in pregnancy
	Spirituality	Thanks giving to God
		Caring for other people
		Trusting in God
